# Construction of a diagnostic signature and immune landscape of pulmonary arterial hypertension

**DOI:** 10.3389/fcvm.2022.940894

**Published:** 2022-12-01

**Authors:** Mengjie Duo, Zaoqu Liu, Yuyuan Zhang, Pengfei Li, Siyuan Weng, Hui Xu, Yu Wang, Tianci Jiang, Ruhao Wu, Zhe Cheng

**Affiliations:** ^1^Department of Respiratory and Critical Care Medicine, The First Affiliated Hospital of Zhengzhou University, Zhengzhou, Henan, China; ^2^Department of Interventional Radiology, The First Affiliated Hospital of Zhengzhou University, Zhengzhou, Henan, China; ^3^Interventional Institute of Zhengzhou University, Zhengzhou, Henan, China; ^4^Interventional Treatment and Clinical Research Center of Henan Province, Zhengzhou, Henan, China

**Keywords:** pulmonary arterial hypertension, weighted gene co-expression network analysis, functional analysis, machine learning, diagnostic model, immune infiltration

## Abstract

**Background:**

Molecular biomarkers are widely used for disease diagnosis and exploration of pathogenesis. Pulmonary arterial hypertension (PAH) is a rapidly progressive cardiopulmonary disease with delayed diagnosis. Studies were limited regarding molecular biomarkers correlated with PAH from a broad perspective.

**Methods:**

Two independent microarray cohorts comprising 73 PAH samples and 36 normal samples were enrolled in this study. The weighted gene co-expression network analysis (WGCNA) was performed to identify the key modules associated with PAH. The LASSO algorithm was employed to fit a diagnostic model. The latent biology mechanisms and immune landscape were further revealed *via* bioinformatics tools.

**Results:**

The WGCNA approach ultimately identified two key modules significantly associated with PAH. For genes within the two models, differential expression analysis between PAH and normal samples further determined nine key genes. With the expression profiles of these nine genes, we initially developed a PAH diagnostic signature (PDS) consisting of LRRN4, PI15, BICC1, PDE1A, TSHZ2, HMCN1, COL14A1, CCDC80, and ABCB1 in GSE117261 and then validated this signature in GSE113439. The ROC analysis demonstrated outstanding AUCs with 0.948 and 0.945 in two cohorts, respectively. Besides, patients with high PDS scores enriched plenty of Th17 cells and neutrophils, while patients with low PDS scores were dramatically related to mast cells and B cells.

**Conclusion:**

Our study established a robust and promising signature PDS for diagnosing PAH, with key genes, novel pathways, and immune landscape offering new perspectives for exploring the molecular mechanisms and potential therapeutic targets of PAH.

## Introduction

Pulmonary arterial hypertension (PAH) is a rapidly progressive and fatal cardiopulmonary disease, and its incidence is about one–two in a million per year (1, 2). The development and progression of PAH are closely associated with structural and functional abnormalities of the pulmonary vasculature. Pulmonary vascular remolding involves intimal injury, middle hypertrophy, adventitia proliferation and fibrosis, and perivascular inflammatory cell infiltration, leading to progressive stenosis and occlusion of the pulmonary artery lumen. Consequently, increased pulmonary vascular pressure results in right heart failure and even death, ultimately, and PAH is characterized by high mortality (3). The gold-standard test for diagnosing PAH is the right heart catheterization (RHC), but the severe complication rate was 1% (4). Although echocardiography is recommended in current guidelines (5), a meta-analysis had elucidated that the pooled sensitivity was 88% (84–92), and specificity was 56% (46–66) for the diagnosis of PAH (6). Besides, the mechanisms of PAH are not understood, especially at the molecular level. Therefore, it is necessary to explore a novel perspective for diagnosing patients with PAH and gaining deeper insights for understanding the biological mechanisms of PAH.

Recently, the rapid development in bioinformatics facilitated the detection of potential biomarkers and the exploration of latent disease mechanisms in PAH. Large-scale research confirmed that the mutations in BMPR2, ACVRL1, ENG, SMAD9, TBX4, KCNK3, and EIF2AK4 in adult-onset patients were related to specific PAH (7). Mutations of multiple genes and aberrant gene expression are involved in the pathogenesis of PAH *via* promoting the proliferation and reducing apoptosis of pulmonary vascular cells. Nevertheless, based on the available discovery, existing biomarkers lack sufficient sensitivity and specificity on account of heterogeneity and confounding factors of samples and the simplicity of the analytical method. Overall, the previous studies are insufficient to interpret the mechanistic pathways of PAH susceptibility and disease progression, and thus, it is essential to detect biomarkers by integrative and insightful analysis between patients with PAH and normal.

In our study, two independent microarray cohorts were generated from the Gene Expression Omnibus (GEO).^1^ In addition, the weighted gene co-expression network analysis (WGCNA), the functional enrichment analysis, and the differentially expressed gene (DEG) analysis were performed to screen the hub genes. Subsequently, the LASSO algorithm was employed to construct a reliable and individualized PAH diagnostic signature (PDS) for diagnosing PAH and evaluating the immune landscape. In addition, the results might shed light on the clinical application and molecular mechanism of PAH.

## Materials and methods

### Data generation and preprocessing

**FIGURE 1:**
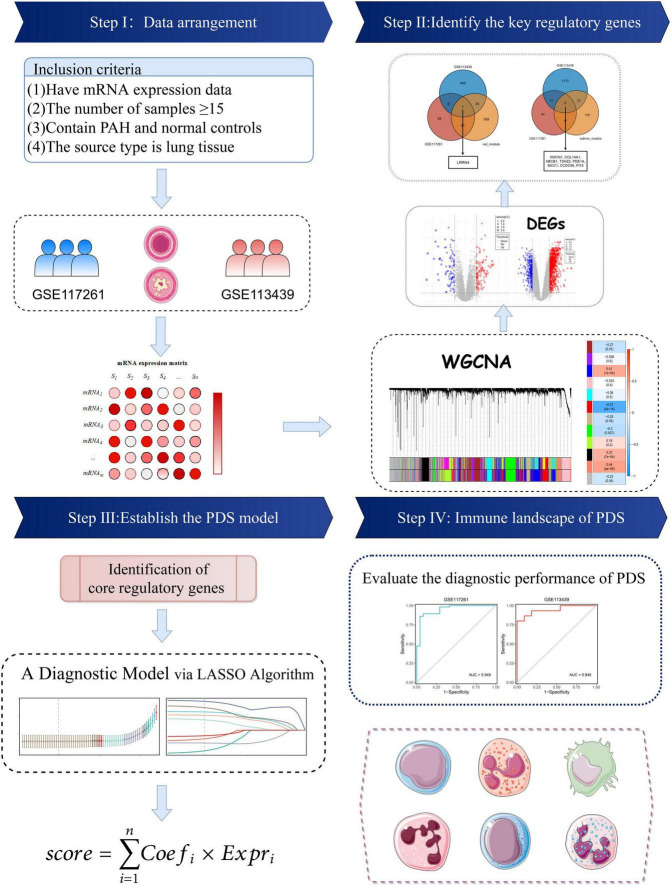
Flowchart of this study.

The keyword “pulmonary hypertension” in GEO’s gene expression profile was searched. Two datasets met the inclusion criteria: (i) the datasets contained complete transcriptome data of PAH and normal lung tissues and (ii) the number of samples was more than ten in each group. The GSE117261 dataset contained total RNA gene expression microarray data from 58 PAH and 25 normal lung tissues (Supplementary Table 1). GSE113439 contains total RNA gene expression microarray data from 15 PAH and 11 normal lung tissues (Supplementary Table 2). They were based on the same platform, GPL6244. The data processing procedure of the research was illustrated in the workflow (Figure 1).

### Co-expression network analysis

The weighted gene co-expression network analysis (WGCNA) was conducted to screen potential modules of high relationship with PAH based on the gene expression profiles *via* the “WGCNA” R package. The expression of genes was ranked *via* standard deviation. Then the top 5,000 genes were picked for the next step of analysis. Next, the hierarchical cluster analysis was used to exclude outlier samples. We calculated the Pearson correlation value between each gene pair to obtain a gene similarity matrix. Then, the formula, aij (adjacency matrix between gene i and j) = | Sij (similarity matrix of all gene pairs)| × β (the soft threshold), was used to construct the adjacency matrix. The optimal β was picked by the “pickSoftThreshold” function in the “WGCNA” R package to meet the scale-free distribution. The adjacency matrix was transformed into a topological overlap matrix (TOM) and a 1-TOM, reflecting the similarity and dissimilarity between genes, separately. Ultimately, the genes were divided into different modules using hierarchical clustering methods. The module eigengene (ME) was calculated, representing the gene expression profile of each module. Therefore, modules highly correlated with PAH were selected as key modules for further analysis. The soft threshold was β = 7, minModuleSize = 50, deep Split = 2, and MEDissThres = 0.3.

### Functional enrichment analysis

The “clusterProfiler” R package was used to further describe potential biological functions and obtain pathways of genes in the WGCNA key gene modules *via* Gene Ontology (GO) enrichment analysis and Kyoto Encyclopedia of Genes and Genomes (KEGG) pathway enrichment analysis. The false discovery rate (FDR) was further computed according to the Benjamini–Hochberg procedure (Benjamini and Hochberg, (8). The FDR < 0.05 was considered as statistically significant.

### Construction of protein–protein interaction (PPI)

To identify the hub genes and PPI network in the key modules, genes within the key modules were further uploaded to the Search Tool for the Retrieval of Interacting Genes (STRING)^2^ for constructing PPI network. The medium confidence score of the PPI network was 0.400. Then the “MCODE” algorithm with default parameters was implemented in the Cytoscape software (version: 3.8.2).

### Differentially expressed gene analysis

The differentially expressed genes (DEGs) between PAH and normal lung tissue were identified through the “limma” R package. *P*-adjusted value < 0.05 and |log2 fold change (FC)| > 2/3 were set as the threshold of DEGs.

### Identification of key regulatory genes

The intersection of the most positive correlation module in the WGCNA and upregulated genes significantly in the two datasets is known as upregulated key genes of PAH. Similarly, the intersection of the most negative correlation module in the WGCNA and downregulated genes significantly in the two datasets is known as downregulated key genes of PAH.

### Machine learning

The least absolute shrinkage and selection operator (LASSO) is a machine-learning algorithm to obtain a robust predictive performance model and is applied to select the best predictive gene for the diagnosis of PAH. This process was achieved through the “glmnet” R package. The performance of PDS was assessed by the area under the receiver operator characteristic (ROC) curve.

### Gene set enrichment analysis

The normalized enrichment score (NES) was calculated for PAH on GO and KEGG pathways in the Molecular Signature Database (MSigDB) *via* all GO gene sets (c5.go.v7.4.symbols.gmt) and KEGG gene sets as gene symbols (c2.cp.kegg.v7.4.symbols.gmt), respectively. | NES| > 1.50, FDR < 0.01, and adjusted *P*-value < 0.01 were set as cutoff criteria.

### Evaluation of immune cell infiltration

To describe the differences in immune cell infiltration between the high-score and low-score groups, we used single-sample gene set enrichment analysis (ssGSEA), which is an extension of GSEA that generates enrichment scores for individual samples. The abundance of infiltrating immune cells was calculated and visualized through the “GSVA” R package (v1.42.0). Furthermore, we evaluated correlation coefficients between PDS scores of samples and immune cell abundance to investigate the main immune cells engaged in the PAH.

### Statistical analysis

The data processing, statistical analysis, and plotting were conducted in R 4.1.0 software. Pearson’s correlation coefficient was assessed for correlations between two continuous variables. The chi-square tests were used to compare categorical variables, while the Wilcoxon rank sum test or *t*-test was used to compare continuous variables. The “survminer” R package was fitted to determine the optimal cutoff value. The LASSO was fitted by “glmnet” R package. The “pROC” R package utilized ROC and the area under the ROC curve (AUC). *P* < 0.05 was determined using the “pROC” R package. It was determined that *P* < 0.05 was statistically significant.

## Results

### Identification of key modules in pulmonary arterial hypertension *via* weighted gene co-expression network analysis

**FIGURE 2:**
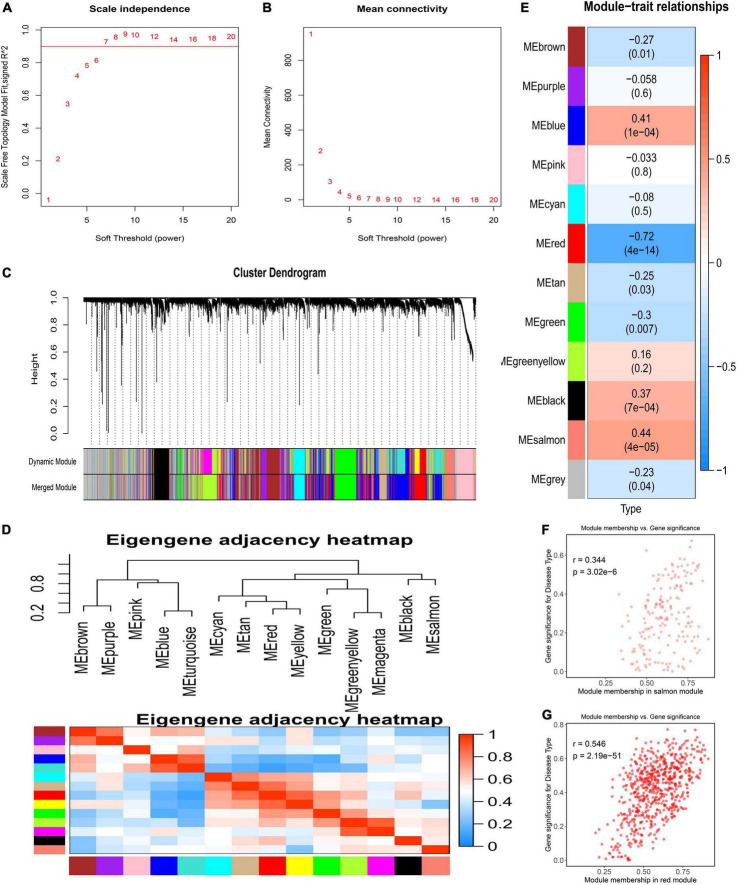
Detection of weighted gene co-expression network and modules. **(A)** Scale-free topological indices at various soft-thresholding powers. **(B)** The correlation analysis between the soft-thresholding powers and mean connectivity of the network. **(C)** Gene clustering diagram based on hierarchical clustering under optimal soft-thresholding power. **(D)** The heatmap of the eigengene adjacency. **(E)** Correlations between gene modules and PAH. **(F)** The correlation between the salmon module memberships and the gene significance. **(G)** The correlation between the red module memberships and the gene significance.

The GSE117261 dataset was used as a training dataset to recognize the key genes associated with PAH. First, two outlier samples were removed, and the 81 samples and the top 5,000 genes were used to obtain the gene similarity matrix. Then, the gene similarity matrix was constructed as an adjacency matrix according to the formula. Second, the soft-thresholding power was set to seven by the “pickSoftThreshold” function for the analysis of a scale-free network (Figures 2A,B). Third, the adjacency matrix was converted to the TOM. We clustered MEs based on calculating the dissimilarity of MEs and using the “mergeCloseModules” function and then 14 MEs were identified (Figures 2C,D). Ultimately, data were visualized in regard to the module–trait relationships based on the Pearson correlation coefficient between the MEs and the disease (Figure 2E). Among these, the salmon module was the top positive module (*r* = 0.441, *P* < 0.0001) with PAH including 646 genes (Figure 2F), and the red module was the top negative module (*r* = –0.718, *P* < 0.0001) including 176 genes (Figure 2G).

### Enrichment analyses and protein–protein interaction construction of key modules

**FIGURE 3:**
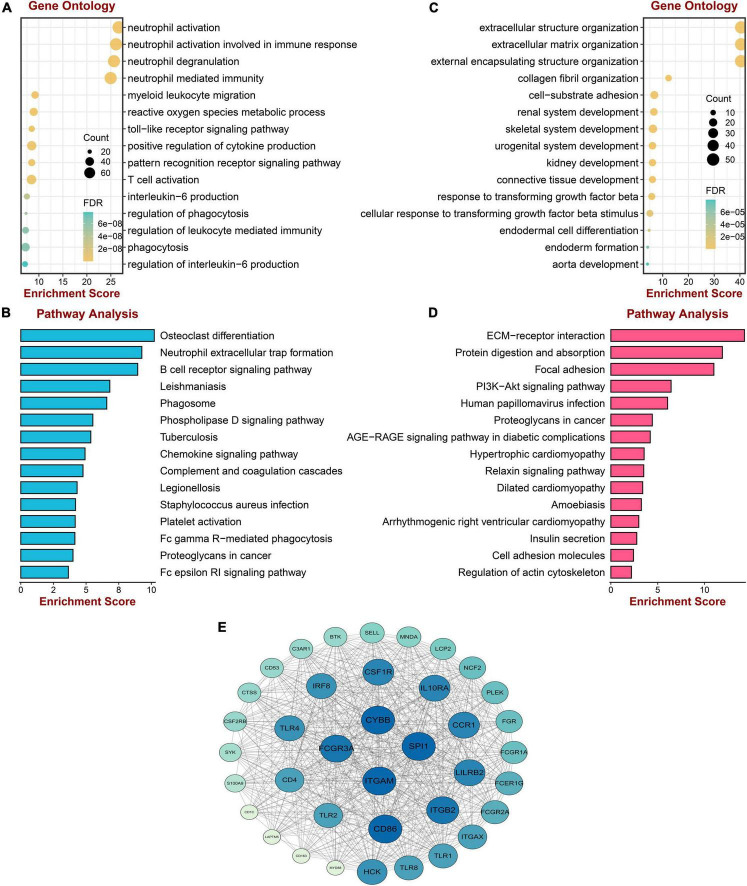
Enrichment analysis and protein–protein interaction construction of key modules. **(A)** GO enrichment analysis of genes in the red module. **(B)** KEGG pathway analysis of genes in the red module. **(C)** GO enrichment analysis of genes in the salmon module. **(D)** KEGG pathway analysis of genes in the salmon module. **(E)** The protein–protein network of two modules.

To acquire a deep understanding of the function of genes in positively and negatively related modules, salmon and red module genes were analyzed through enrichGO and enrichKEGG function in the “clusterProfiler” R package, respectively. Genes of the red module were significantly enriched in “neutrophil activation,” “neutrophil activation involved in immune response,” “neutrophil degranulation,” and “neutrophil mediated immunity,” all of which were terms about neutrophil, as shown in Figure 3A. The KEGG pathway terms were related to “Osteoclast differentiation,” “Neutrophil extracellular trap formation,” and “B cell receptor signaling pathway,” which might play essential roles in PAH (Figure 3B). Meanwhile, the top three GO terms were enriched by genes of the salmon module, including “Extracellular structure organization,” “Extracellular matrix organization,” and “External encapsulating structure organization,” which were mainly associated with the extracellular organization (Figure 3C). The KEGG pathways suggested that the “ECM–receptor interaction” and “protein digestion and absorption” might be potential pathways of PAH (Figure 3D). The lists of genes involved in the GO and KEGG enrichment analysis in red and salmon modules can be found in Supplementary Tables 3–6. These results indicated that inflammation and immune cells played a significant role in the process of PAH.

To screen the hub gene of positive and negative correlation modules with PAH, the PPI network was established through the STRING database, including 852 nodes and 5,841 edges. Then the network was processed in the Cytoscape software, and the possible 38 essential genes ranked by node degree were visualized using the MCODE plugin. The top 10 highest degrees of genes were screened, including ITGAM, CYBB, SPL1, FCGR3A, CD86, ITGB2, LILRB2, CCR1, 1L10RA, and CSF1R (Figure 3E).

### Identification of differentially expressed genes in two pulmonary arterial hypertension datasets

**FIGURE 4:**
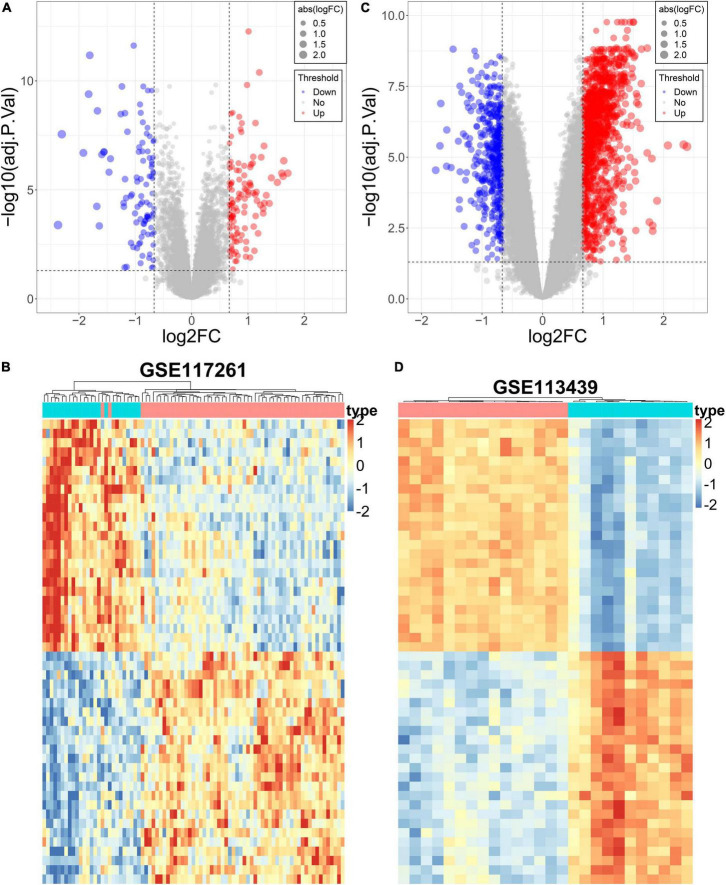
Differential expression analysis of the PAH datasets. **(A)** The volcano plot of DEGs in GSE117261. **(B)** The heatmap of DEGs in GSE117261. **(C)** The volcano plot of DEGs in GSE113439. **(D)** The heatmap of DEGs in GSE113439.

The DEGs in lung tissue between the patients with PAH and normal controls were excavated by the “limma” R package. Consequently, in the GSE113439 dataset, 1,355 significantly upregulated genes and 483 significantly downregulated genes were defined. Similarly, in the GSE117261 dataset, we identified 120 significantly upregulated genes and 99 significantly downregulated genes. These DEGs are shown as a volcano plot and heatmap in Figures 4A–D.

### Determination of the key genes

**FIGURE 5:**
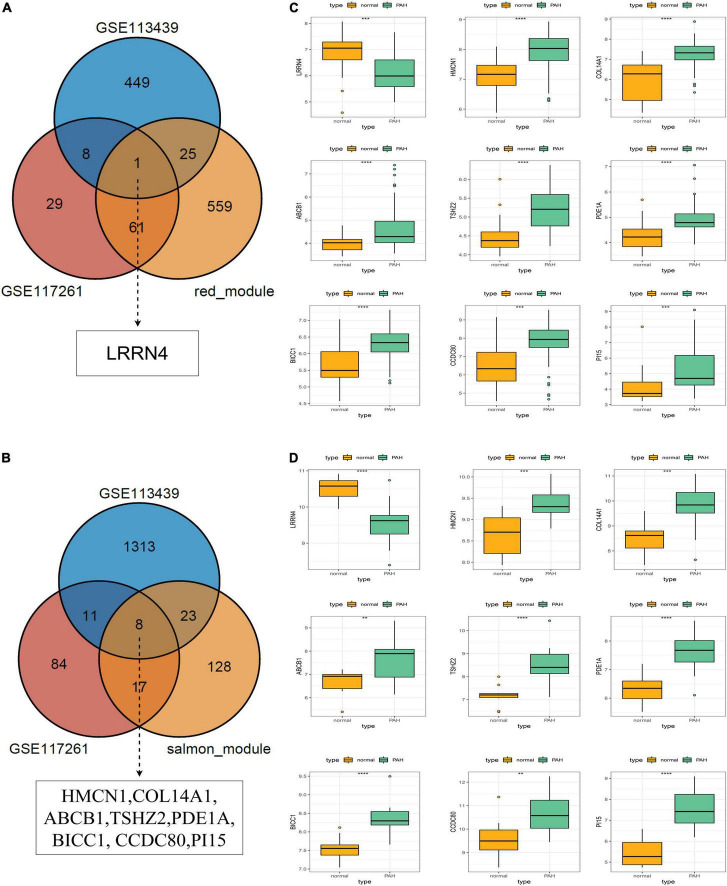
Selection and validation of key regulatory genes. **(A)** Venn diagram to indicate one downregulatory gene from the red module and DEGs. **(B)** Venn diagram to indicate eight upregulatory genes from the salmon module and DEGs. **(C)** Validation of key regulatory genes in the dataset GSE117261. **(D)** Validation of key regulatory gene in the dataset GSE113439.

The core downregulated gene was screened through the intersection of the genes in the red module of WGCNA and the significantly downregulated genes in two datasets, including LRRN4 (Figure 5A). Likewise, the key upregulated gene was screened through the intersection of the genes in the salmon module of WGCNA and the significantly upregulated genes in the two datasets, including PI15, BICC1, PDE1A, TSHZ2, HMCN1, COL14A1, CCDC80, and ABCB1 (Figure 5B). The expression levels of nine key genes are verified in the two datasets shown in Figures 5C,D.

### Construction of a diagnosis model *via* least absolute shrinkage and selection operator algorithm

**FIGURE 6:**
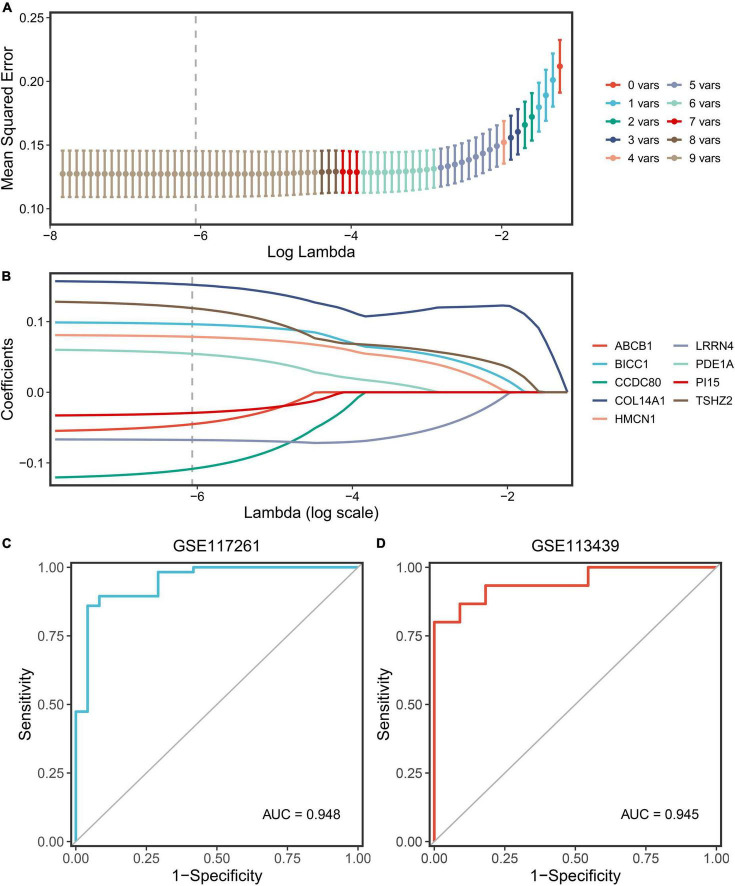
Screening and validation of the genes. **(A)** Determination of the optimal lambda was obtained when the partial likelihood deviance reached the minimum value and further generated the key gene with non-zero coefficients. **(B)** LASSO coefficient profiles of the candidate gene for PDS construction. **(C)** The ROC curve of the modeling dataset (GSE117261). **(D)** The ROC curves of validation datasets (GSE113439).

The nine key genes were developed as a reliable and individualized PAH diagnostic signature (PDS) by applying the LASSO algorithm to diagnose patients with PAH. The optimal lambda was 0.002116 when the LASSO regression partial likelihood deviation was minimized (Figure 6A). Consequently, nine key genes with non-zero LASSO coefficients were regarded as the diagnostic model’s main variables (Figure 6B). The nine genes were COL14A1, TSHZ2, CCDC80, BICC1, HMCN1, LRRN4, PDE1A, ABCB1, and PI15, and their coefficients were 0.1522, 0.1191, –0.1084, –0.0963, 0.0785, –0.0676, –0.0545, –0.0452, and –0.0292, respectively. The ROC analysis demonstrated outstanding AUCs with 0.948 and 0.945 in two cohorts for evaluating the power of the PDS to differentiate the PAH (Figures 6C,D). Therefore, we established an optimal diagnostic signature PDS with the formula: PDS score = 0.1522 × Exp COL14A1 + 0.1191 × Exp TSHZ2 - 0.1084 × Exp CCDC80 - 0.0963 × Exp BICC1 + 0.0785 × Exp HMCN1 - 0.0676 × Exp LRRN4 - 0.0545 × Exp PDE1A – 0.0452 × Exp ABCB1 - 0.0292 × Exp PI15 + 0.7037.

### Exploration of biological mechanisms *via* gene set enrichment analysis (GSEA)

**FIGURE 7:**
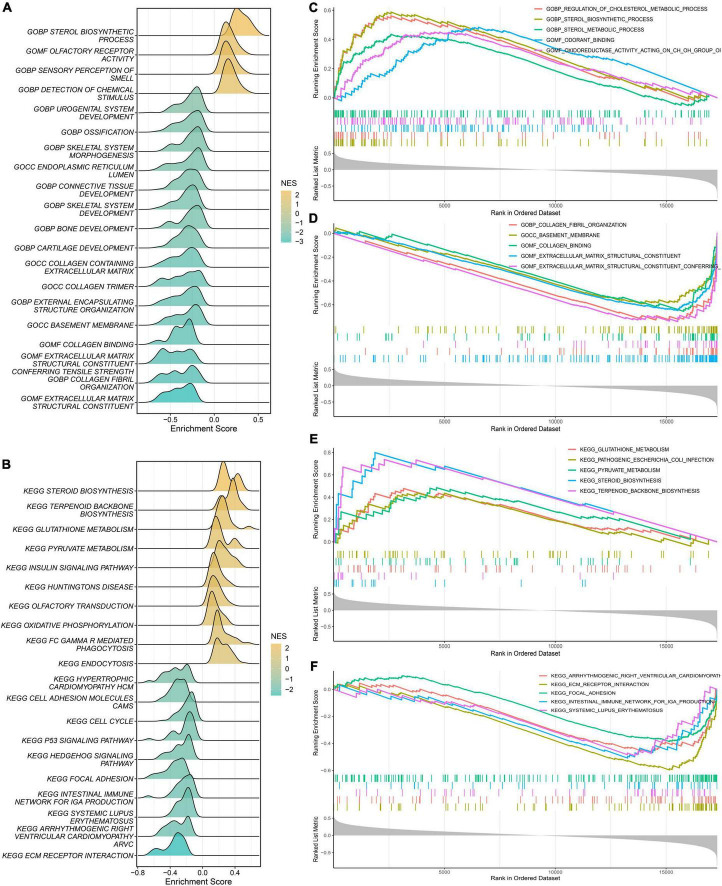
Gene Set Enrichment Analysis (GSEA). **(A)** The ridge plot of the top 20 GO terms with ranked genes of the modeling dataset. **(B)** The ridge plot of the top 20 KEGG pathways with ranked genes of the modeling dataset. **(C,D)** The positive and negative top five GO terms with ranked genes of the modeling dataset. **(E,F)** The positive and negative top five KEGG pathways with ranked genes of the modeling dataset.

First, we calculated the PDS scores and gene expression correlations for gene sequencing. Subsequently, GSEA was performed to detect potential mechanisms for PAH. Figures 7A,B illustrate the most important GO terms and the KEGG pathways. Among these, Figure 7C depicts the top five positively relevant GO terms, including “Regulation of cholesterol metabolic process,” “Sterol biosynthetic process,” “Sterol metabolic process,” “Odorant binding,” and “Oxidoreductase activity acting on CH-OH group.” Figure 7D depicts the top five negatively relevant GO terms, comprising “Collagen fibril organization,” “Basement membrane,” “Collagen binding,” “Extracellular matrix structural constituent,” and “Extracellular matrix structural constituent conferring.” On the contrary, Figure 7E describes the top five positively correlated the KEGG pathways, consisting of “Glutathione metabolism,” “Pathogenic *Escherichia coli* infection,” “Pyruvate metabolism,” “Steroid biosynthesis,” and “Terpenoid backbone biosynthesis.” Likewise, Figure 7F describes the top five negatively correlated KEGG pathways, consisting of “Arrhythmogenic right ventricular cardiomyopathy ARVC,” “ECM-receptor interaction,” “Intestinal immune network for IgA production,” and “Systemic lupus erythematosus.” Notably, “ECM–receptor interaction” was enriched once again which was enriched in the salmon KEGG pathway. It can be concluded that the “extracellular matrix organization” may play an essential role in PAH.

### Immune landscape of PAH diagnostic signature

**FIGURE 8:**
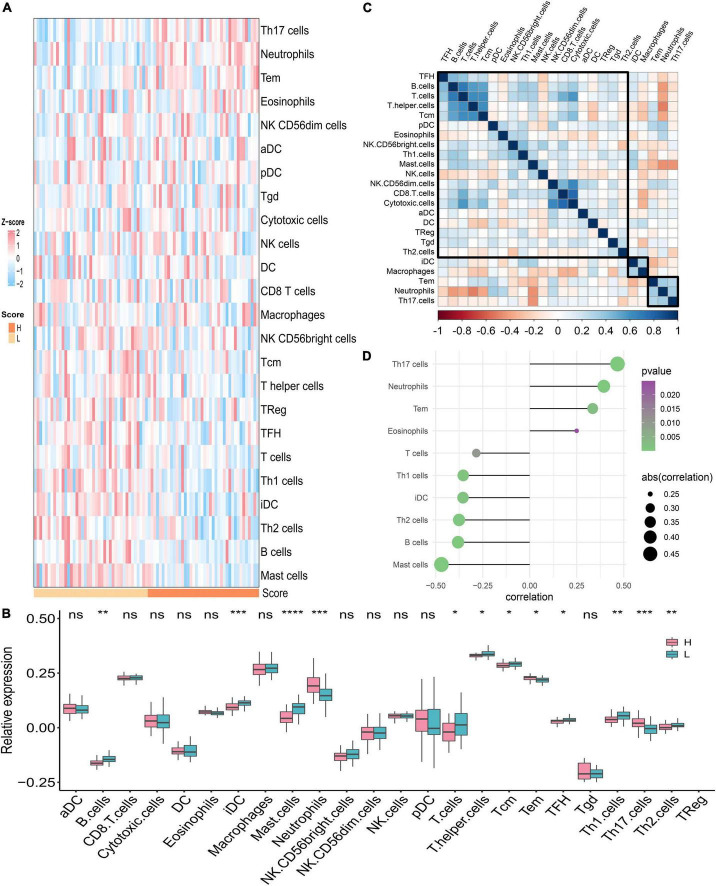
Analysis of immune landscape associated with PAH. **(A)** The heatmap of the immune infiltration in high- and low-score groups. **(B)** The boxplot of the 24-type immune infiltration in high- and low-score groups. *P < 0.05, **P < 0.01, and ***P < 0.001. **(C)** The heatmap of the correlations between different immune cells. **(D)** Relationship between the PDS score and immune infiltration. H: high score; L: low score. aDC: activated dendritic cell; DC: dendritic cell; iDC: interdigitating cell; NK cells: natural killer cells; pDC: plasmacytoid dendritic cells; Tcm: central memory T cell; Tem: effector memory T cell; TFH: T follicular helper cell; Tgd: gamma delta T cells; TReg: T regulatory cells.

We assumed that the two PDS score groups had different immunological characteristics since inflammation and immune cells are essential in the PAH process. To probe the discriminating immune landscape of patients with PAH, the ssGSEA algorithm was used to estimate the infiltration abundance of 24 types of immune cells among the GSE117261 dataset. The fraction of 24 types of immune cells in GSE117261 samples is depicted as a heatmap in Figure 8A. The relative expression is portrayed as a boxplot in Figure 8B. We can see that a superior abundance of Th17 cells, neutrophils, effective memory T cell (tem), and eosinophils was the immune feature of the high-score group, whereas high infiltration of mast cells, B cells, Th2 cells, interdigitating cell (iDC), Th1 cells, and T cells was the immune feature in the low-score group. Figure 8C shows the heatmap of correlations between immune cells. The T cells and B cells showed the strongest positive correlation, and the neutrophils and T helper cells showed the strongest negative correlation. Subsequently, we probed the correlation between the PDS score and immune infiltration. As shown in Figure 8D, the infiltration level of Th17 cells (*r* = 0.467, *P* < 0.0001), neutrophils (*r* = 0.394, *P* = 0.0003), tem (*r* = 0.335, *P* = 0.0023), and eosinophils (*r* = 0.250, *P* = 0.0249) was positively correlated with the PDS score; the infiltration level of mast cells (*r* = –0.470, *P* < 0.0001), B cells (*r* = –0.381, *P* = 0.0005), Th2 cells (*r* = –0.376, *P* = 0.0006), iDC (*r* = –0.355, *P* = 0.0012), Th1 cells (*r* = –0.354, *P* = 0.0012), and T cells (*r* = –0.284, *P* = 0.0103) was negatively associated with the PDS score.

## Discussion

Pulmonary arterial hypertension is a disorder characterized by a progressive increase in pulmonary vascular stress and significant pulmonary vascular remodeling, resulting in hypertrophy and remodeling of the right ventricle (9–11). If a patient with pulmonary hypertension is not diagnosed promptly, the probability of death due to right ventricular failure is drastically increased (9). As a rapidly evolving approach, molecular analysis is utilized to comprehend the latent pathways in the context of human disease. The notion is recognized that PAH is actuated by a comprehensive network of molecular processes (12–15). Measurement of RNA expression is one of the high-throughput unbiased techniques in the Omics approach, which provides a snapshot of the transcriptome aspect (13). These insights provide new perspectives for predicting potential pathogenesis and therapeutic aspects. Therefore, it is of great significance to explore molecular biomarkers and to construct a diagnostic model for the diagnosis of PAH.

The WGCNA, as a bioinformatics approach, explicitly exploits the relationship between gene co-expression modules and disease to further explore the pathogenesis of diseases. Our study screened out 11 modules associated with PAH *via* WGCNA. Among the 11 modules screened, the genes in the salmon module are the most positively correlated with PAH and those in the red modules are the most negatively correlated with PAH. In the salmon modules, genes were mainly concentrated in extracellular structure organization, extracellular matrix (ECM) organization, and external encapsulating structure organization in GO terms and gathered in the KEGG pathway of ECM–receptor interaction and PI3K–Akt signaling pathway. As mentioned in the literature review, ECM remodeling triggers pulmonary arterial smooth muscle proliferation and pro-inflammatory response in the endothelial cells resulting in increased stiffness of the proximal and distal pulmonary arteries in PAH (16, 17). Meanwhile, the PI3K–Akt signaling pathway is an essential nexus of pulmonary artery smooth muscle cell (PASMC) proliferation and hypoxia-induced pulmonary vascular remodeling (18, 19). Conversely, in the red modules, genes were mainly concentrated in GO terms related to neutrophil and immune response. Besides, genes were gathered in the B-cell receptor signaling pathway and neutrophil extracellular trap (NET) formation in the KEGG pathway. The release of neutrophil elastase is part of neutrophil activity. NE, which is found in PASMCs and neointimal lesions of PAH, is thought to cause vascular remodeling by causing the release of growth factors, aggregation and activation of their receptors, and subsequent migration and proliferation of smooth muscle cells and fibroblasts through extracellular matrix degradation (20–22). NETs, formed from chromatin decondensation provoked by reactive oxygen species (ROC), can trigger the inflammatory activation of lung endothelial cells and stimulate endothelial angiogenesis through myelo-peroxidase/H2O2/NFkB/TLR4-dependent signaling. These results confirm the findings of extensive previous work demonstrating the potential pathological relevance between NETs and inflammatory angiogenesis, a disturbance of vascular homeostasis in PAH (23). In summary, the comprehensive bioinformatics analysis perceived that neutrophil activation and immune response played a considerable role in disease pathogenesis and the process of PAH. On the contrary, the complexity of cytokine, cellular immunity, and autoantibody changes indicated that PAH might be an autoimmune and inflammatory disease, which was consistent with previous reports (11, 24–26).

From a broad perspective, the development of PDS for clinical application is of great significance. Previous studies have examined key genes by DEG analysis between PAH lung specimens and normal lung specimens solely based on the public database in the GEO (27–29). The presence of heterogeneity of the disease and confounding factors reduces the sensitivity and specificity of DEGs as biomarkers for PAH. In addition, redundant key genes limited the clinical practice of the clinical application. In our study, the essential biomarkers relevant to the PAH were filtrated by combining the results of the WGCNA and the DEGs. Further analysis identified nine robust PDS by the application of the LASSO algorithm, including COL14A1, TSHZ2, CCDC80, BICC1, HMCN1, LRRN4, PDE1A, ABCB1, and PI15. The PDS demonstrated high discriminatory power with outstanding AUCs in the two cohorts separately. Phosphodiesterase 1 (PDE1), encoded by three genes, namely PDE1A, 1B, and 1C, is a sub-family of enzymes. Some animal studies have shown that the inhibition of PDE1A treats pulmonary arterial hypertension by reversing pulmonary vascular remodeling and right heart hypertrophy (30, 31). The basement membrane collagen COL4A5 was significantly upregulated in the intima and media of the IPAH patient cohort, indicating improved vascular stiffness *via* stabilizing existing collagen fibers (32). PI15 belongs to the CAP superfamily of proteins and is a trypsin inhibitor (33). Against extracellular matrix proteins, trypsin has high protease activity. PI15 has been hypothesized to perform a protective role in elastic tissues against proteolytic damage and a role in controlling extracellular matrix changes (34). LRRN4, also known as leucine-rich repeat neuron protein-4, is a member of the LRRN family and linked to a range of pathological events, including cardiac remodeling (35, 36). BICC family RNA-binding protein 1 (BICC1) is an RNA-binding protein that modulates protein translation to control gene expression. BICC1 can influence biological processes including proliferation and apoptosis. Furthermore, abnormal BICC1 expression has been linked to immune cell infiltration during disease progression (37). Hemicentin-1 (HMCN1) is an ECM fibulin protein that is thought to be required for stable cell-to-cell interactions and ECM structure stability and may interact with receptors on the cell surface, either directly or indirectly, providing a mechanism for cell behavior modulation (38, 39). Taken together, exploring the underlying mechanisms in PAH of key genes contained in PDS might facilitate the clinical translation and application of the diagnostic model.

The PDS score-based GSEA indicated that immune-related pathways were enriched between high and low groups. Hence, deciphering the exact mechanisms of immune cells in pulmonary vessels might lead to a wide range of potential attractive therapeutic targets for PAH therapy. We further estimated the fraction of 24 immune cells between the two groups *via* the ssGSEA algorithm. We discovered that the Th17 cells, neutrophils, tem, and eosinophils were at high expression in the high-score subgroup compared to the low-score subgroup, while mast cells, B cells, Th2 cells, iDC, Th1 cells, and T cells presented low infiltration levels in the low-score group. Th17 cells, a subpopulation of effector T cells that produce IL-17, are highly pro-inflammatory and are widely involved in inflammatory diseases (40). It has been shown that IL-17 is of significance in chronic inflammation-associated pulmonary hypertension, where it correlates with disease severity in SSc-associated pulmonary hypertension. Neutrophils release NE, present in PASMCs of PAH, which can lead to vascular remodeling through aggregation and activation of growth factors and their receptors, and degradation of the smooth muscle cell and fibroblast migration and proliferation (21). In addition, there is growing evidence that eosinophil infiltration of the pulmonary vasculature is an important, influential factor in the pathological changes of all types of PAH (41). Eosinophils stimulate pulmonary vascular remodeling by releasing granular content and stimulating intravascular PASMC proliferation. Combined with previous studies, we further confirmed that abnormal immune cell expression was critical in the pathogenesis of vascular remodeling and might be potential targets for PAH treatment.

Although advanced bioinformatics techniques and machine-learning algorithms are combined to identify candidate genes and construct diagnostic models for PAH, several limitations should be noticed. First, the relevant genes and pathways screened are not experimentally validated. Fundamental study validation is required for better clinical application in further studies. Second, the PDS needs to be validated with a larger sample size. Last, the dataset lacked comprehensive information on clinical aspects.

In summary, our study constructed a nine-gene diagnostic model of PAH and PDS, through comprehensive bioinformatics analysis. Two modules significantly associated with PAH were identified, and key genes and novel mechanistic pathways were identified. In addition, the inflammatory and immune landscapes of patients with PAH were depicted. Overall, the key genes, novel pathways, and immune landscape may shed light on exploring the molecular mechanisms and potential therapeutic targets of PAH.

## Data availability statement

The datasets presented in this study can be found in online repositories. The names of the repository/repositories and accession number(s) can be found in the article/Supplementary material.

## Author contributions

MD, ZL, and ZC designed the research. MD, ZL, YZ, and SW performed the data acquisition and data analysis. HX assisted with data analysis. MD and PL wrote the manuscript. YW, TJ, and RW edited and revised the manuscript. All authors read and approved the manuscript.

## Footnotes

1
http://www.ncbi.nlm.nih.gov/geo/

2
https://string-db.org/
